# The Combined Use of *Pediococcus pentosaceus* and Fructooligosaccharide Improves Growth Performance, Immune Response, and Resistance of Whiteleg Shrimp *Litopenaeus vannamei* Against *Vibrio parahaemolyticus*

**DOI:** 10.3389/fmicb.2022.826151

**Published:** 2022-02-25

**Authors:** Nguyen Thi Xuan Hong, Nguyen Thi Hue Linh, Kartik Baruah, Do Thi Bich Thuy, Nguyen Ngoc Phuoc

**Affiliations:** ^1^Faculty of Fisheries, University of Agriculture and Forestry, Hue University, Hue, Vietnam; ^2^Department of Animal Nutrition and Management, Aquaculture Nutraceuticals Research Group, Faculty of Veterinary Medicine and Animal Sciences, Swedish University of Agricultural Sciences, Uppsala, Sweden; ^3^Faculty of Engineering and Food Technology, University of Agriculture and Forestry, Hue University, Hue, Vietnam

**Keywords:** *P. pentosaceus*, fructooligosacharide, *L*. *vannamei*, growth performance, immune response

## Abstract

In this study, we evaluated the effect of probiotic bacteria *Pediococcus pentosaceus* supplemented at different inclusion levels in a control diet [basal diet containing 0.5% fructooligosaccharide (FOS)] on the growth performance, feed conversion ratio, immune response, and the disease resistance of whiteleg shrimp *Litopenaeus vannamei* juveniles against *Vibrio parahaemolyticus*. A control diet with 0.5% FOS but without *P. pentosaceus* supplementation (Control) was prepared. In addition, three other test diets were also formulated: control diet supplemented with *P. pentosaceus* at (i) 1 × 10^6^ cfu g^–1^ diet (P1), (ii) 1 × 10^7^ cfu g^–1^ diet (P2), or (iii) 1 × 10^8^ cfu g^–1^ diet (P3). After a 60-day feeding trial, the experimental shrimps were challenged with *V. parahaemolyticus.* The results showed that dietary supplementation of *P. pentosaceus* significantly improved the growth performance and immune responses of *L. vannamei* juveniles. The juveniles that were fed with a P2 or P3 diet recorded the maximum increase in the final body weight, final length, weight gain, and survival rate. The total hemocyte counts, phenoloxidase, and lysozyme activity of shrimp fed with either of these two diets were significantly enhanced. The results also showed that juveniles fed with a P2 or P3 diet exhibited significantly lower mortality when challenged with *V. parahaemolyticus*. Overall results suggested that a combination of *P. pentosaceus* at the inclusion level of 1 × 10^7^ cfu g^–1^ diet (P2) and 0.5% FOS could be considered as a potential synbiotic formulation for improving the growth, health, and robustness of *L. vannamei*.

## Introduction

Aquaculture accounted for 46% of the total global fish production and 52% of fish for human consumption (FAO, 2020). It plays an important role in providing the growing population with high-quality animal protein, and creating job all over the world, including in Vietnam and many Southeast Asian countries. The whiteleg shrimp *Litopenaeus vannamei* is one the most popular cultured penaeid species in Asian countries, reaching 5.4 million tons in 2019 (FAO, 2021). Diseases outbreak caused by viral and bacterial pathogens has been the main obstacle to the sustainable production of this species both in Asia and in other parts of the world. In particular, the outbreaks of acute hepatopancreatic necrosis disease (AHPND) in farmed marine shrimp, which is caused by virulent strains of bacteria belonging to *Vibrio parahaemolyticus* and related species, caused mass mortalities in cultured *L. vannamei* and giant tiger prawn (*Penaeus monodon*) (Shinn et al., 2018; Tang et al., 2020). The economic losses due to this disease have amounted to over USD 7 billion annually (Tang et al., 2020), and therefore AHPND was listed by the World Organization for Animal Health (OIE) as a notifiable disease in 2019 (OIE, 2019).

The application of antibiotic has been an effective strategy for preventing an extensive range of Gram-negative/positive bacteria in farmed shrimp (Baticados and Paclibare, 1992; Yano et al., 2014). However, the indiscriminate use of antibiotics in shrimp aquaculture has led to the rapid development of resistant bacterial strains, which eventually constitute a direct threat to human health and to the environment (Cabello, 2006; DeLorenzo et al., 2016; Knipe et al., 2021). Antimicrobial resistance (AMR) is currently one of the most important human and farmed animal health-threatening issues worldwide (Watts et al., 2017; Okocha et al., 2018). Consequently, there is an urgent need to develop a strategy to prevent pathogen propagation and enhance shrimp immunity during farming operations. Probiotics are defined as live microorganisms that confer a health benefit on the host when administered in adequate amounts (Hill et al., 2014). Over the past few years, probiotics are becoming increasingly popular alternatives to antibiotics for promoting growth and health status and preventing disease in shrimp aquaculture. Several studies have shown the important role of probiotics in the competitive exclusion of pathogenic bacteria, nutrient, and enzymatic contribution to shrimp digestion, enhancement of the shrimp immune response, and antiviral effects (Hoseinifar et al., 2018; Ringø, 2020). Probiotics can be administered orally with the feed, or directly into the shrimp rearing water as purified cultures or spores (Ringø, 2020), or in a fermented growth media, for example, *Bacillus subtilis* E20-fermented soybean meal (Tsai et al., 2019; Wang et al., 2019). Similarly, probiotics may be administered in combination with a non-digestible food ingredient (i.e., prebiotics) that beneficially affects the host by causing synergistic effects, an approach referred to as “synbiotics” (Schrezenmeir and de Vrese, 2001; Li et al., 2018; Knipe et al., 2021). The synbiotic formulation comprised of evaluated probiotics corresponds to two bacterial genera, *Enterococcus* spp. and *Bacillus* spp., with prebiotics, such as mannan oligosaccharide (MOS) and fructooligosaccharide (FOS), and has been studied on fish species, such as rainbow trout, Japanese flounder, yellow croaker, and cobia (Cerezuela et al., 2011; Huynh et al., 2017; Villumsen et al., 2020). Synbiotic improves the survival, disease resistance, and microbial ecology of the gastrointestinal tract of the host leading to greater benefits than the application of individual probiont (Ohtani et al., 2020. Knipe et al., 2021). *Pediococcus pentosaceus*, a lactic acid bacteria species, has been demonstrated as a promoter of growth, immune response, and disease resistance, while replacing antibiotics in farmed shrimp (Leyva-Madrigal et al., 2011; Adel et al., 2017; Won et al., 2020). The supplementation of FOS in the feed has proven beneficial for improving growth performance, immune response, and survival of whiteleg shrimp (Hu et al., 2019; Mustafa et al., 2020). However, the modes of action of *P. pentosaceus* combined with FOS on the host’s immune responses are still unclear (Cerezuela et al., 2011; Huynh et al., 2018). This study aims to examine whether the growth performance, immune response, and resistance towards *V. parahaemolyticus* challenge could be improved when the *L. vannamei* were fed diets supplemented with a combination of *P. pentosaceus* and FOS.

## Materials and Methods

### Preparations of Probiotic

The selected *P. pentosaceu*s R6 evaluated in this study was isolated from home-made fermented anchovy sauce, a commercial fish sauce product in Thua Thien Hue province, Vietnam according to the modified method of Adel et al. (2017). The fish sauce was rinsed, homogenized, and suspended in sterile phosphate buffer saline (PBS) at a ratio of one fish sauce and 9 PBS (v/v). Then, the suspension was serially diluted to 10^–3^, 10^–4^, 10^–5^, and 10^–6^. Finally, the suspension was plated on the De Man Rogosa and Sharp (MRS) agar (Merck, Damstadt, Germany) and was incubated under the aerobic condition at 28°C for 24 h. Among 11 strains isolated from the anchovy fish sauce, six isolates were identified as *P. pentosaceu*s according to the results of 16S rDNA sequencing using the general-purpose primers of prokaryotic 16S rDNA: 27F (5′-AGAGTTTGATCCTGGCTCAG-3′), and 1492R (5′-AAGGAGGTGATCCAGCCGCA-3′) (Lane, 1991). The six isolates of *P. pentosaceu*s were tested for antagonistic effect to *V. parahaemolyticus* by using the agar-diffusion method (Bernal et al., 2015) and hemolysis activity in whiteleg shrimp blood according to the description of Chang et al. (2000). Briefly, the *P. pentosaceus* isolates were grown on MRS broth for 24 h at 28°C. One hundred microliters of *V. parahaemolyticus* suspension at a concentration of 10^6^ cfu ml^–1^, which was recovered from AHPND outbreaks in Thua Thien Hue province (Vietnam), was plated on the TSA (Tryptone Soya Agar, Oxoid, Hamsphire, United Kingdom) with 2% NaCl added. After 30 min, from the TSA plate, six 2-mm agar plugs were removed by a cork borer (2 mm diameter) and 100 μl of overnight culture of each *P. pentosaceus* isolate was placed on these wells. The plates were then incubated at 28°C for 24 h and the diameter of the zone of inhibition was measured for determining the antagonistic activity.

A shrimp blood agar consisting of 1 ml of whiteleg shrimp hemolymph in medium containing 200 ppm Rose Bengal was used to determine the hemolytic activity of 6 isolates of *P. pentosaceus* (Chang et al., 2000). Hemolymph (1 ml) was drawn between the first and second pleopods from whiteleg shrimp previously surface-disinfected with 70% ethanol. The hemolymph (1 ml) was immediately transferred into a sterilized tube containing 0.2 ml of citrate-EDTA buffer (0.1 M glucose, 30 mM trisodium citrate, 26 mM citric acid, and 10 mM EDTA dissolved in 20 ppt seawater) and stained by the addition of 133 μl of 3% (w/v) Rose Bengal (dissolved in citrate EDTA buffer) with gentle rotation to achieve a complete mixture. Rose Bengal-stained hemolymph (1 ml) was added to 15 ml of the Nutrient Agar (Himedia, Mumbai, India) with l.5% NaCl, which cooled to 45–50°C in a water bath with gentle shaking for proper mixing and pouring into petri dishes. The six isolates of *P. pentosaceus* were inoculated onto the shrimp blood agar plate. The plates were incubated for 48 h at 28°C and observed for the formation of a transparent zone around the inoculated colonies.

Based on the results of the antagonistic effect and non-hemolytic (see Supplementary Data) on whiteleg shrimp blood agar, the *P. pentosaceu*s R6 isolate was selected for preparing probiotic. The *P. pentosaceus* R6 was cultured and incubated at 28°C for 48 h on MRS agar. From a pure bacterial growth plate of *P. pentosaceus* R6 on MRS agar, a single colony was isolated and placed directly into 20 ml of sterile MRS broth and incubated for 48 h at 28°C in the shaking incubator (Kuhner shaker, ISF-1-W, Switzerland; 140 rpm). After 48 h, the bacterial suspension was centrifuged at 3,500 rpm (Sanyo NSE Mistral 2000R, Japan) for 30 min, washed twice in sterile PBS containing 0.02 M phosphate and 0.15 M NaCl, and the cell pellets obtained were re-suspended in PBS to achieve an OD_600nm_ value of 1, which was equivalent to 1 × 10^9^ cfu ml^–1^ based on standard bacterial growth curves (data not presented). The actual bacterial concentration was determined by viable colony counts according to the method of Miles et al. (1938). The pellets were collected by centrifugation at 3,500 rpm for 30 min, mixed with 20% skimmed milk, and stored at −70°C. The frozen bacterial sample was concentrated in a freeze-dryer and homogenized, followed by storage of the bacterial powder at 4°C until used. The viability of the bacterial mixture was determined according to the method of Miles et al. (1938).

### Preparations of Experimental Diets

In total, one control and three test diets were formulated in this study. Feed formulation and proximate composition of the control diet prepared with the supplementation of 0.5% FOS in the basal diet are shown in Table 1. The control diet was not supplemented with *P. pentosaceus* (Control), whereas the three other test diets were formulated prepared by supplementing the control diet with *P. pentosaceus* at the inclusion level of 1 × 10^6^ cfu g^–1^ diet (P1), 1 × 10^7^ cfu g^–1^ diet (P2), or 1 × 10^8^ cfu g^–1^ diet (P3). The preparation and storage of the diets were conducted following Adel et al. (2017). All the dry ingredients were weighed, ground in a hammer mill to pass through a 250-μm-mesh screen, and then mixed in a mixing machine. After that, fish oil and water were added until a dough was formed. Each diet was then passed through a laboratory pelleting machine (3A, Vietnam) to obtain pellets of size 0.5 to 1 mm diameter. The pellets were air-dried for 72 h to a moisture level of <10%, and then stored at 4°C in the refrigerator until use. New batches of feed were produced every 2 weeks to keep up *P. pentosaceus* viability.

**TABLE 1:** Ingredients and composition (% dry weight basis) of the control diet for whiteleg shrimp used in this study.

Ingredient	g kg^–1^
Fish meal^1^	350.0
Soybean meal^1^	200.0
Wheat meal^1^	285.0
Lecithin^1^	20.0
Fish oil^1^	30.0
Vitamin mixture^2^	20.0
Mineral mixture^2^	20.0
Calcium dihydrogen phosphate^3^	25.0
Cholesterol	25.0
Corn starch^1^	20.0
Fructooligosaccharide (FOS)	5.0

**Proximate composition**	**%**

Moisture (g kg^–1^)	11.5
Protein (g kg^–1^ dry matter)	40.2
Lipid (g kg^–1^ dry matter)	7.3
Ash (g kg^–1^ dry matter)	12.3

*^1^The GreenFeed Co., Ltd., Vietnam.*

*^2^Vemedim (Vietnam). Premix detailed by Won et al. (2020), ^3^Sigma-Aldrich, Vietnam.*

### Shrimp and Experimental Design

Juveniles of *L. vannamei* were purchased from The National Breeding Centre for Central Aquaculture at Phu Hai Commune, Phu Vang district, Thua Thien Hue province, Vietnam. They were transferred by air-conditioned car to the wet lab of Fish Pathology Laboratory of the University of Agriculture and Forestry, Hue city, Vietnam. The juveniles were maintained in 1,000-L fiberglass tanks using continuous flow-through water at 0.38 L min^–1^ at 28 ± 2°C. They were acclimatized to the experimental conditions for 14 days during which they were fed four times a day with the control diet. Shrimp used in this study were quarantined for free AHPND, white spot disease, and yellow head disease at Veterinary Clinic, sub-department of Animal Husbandry and Veterinary Medicine in Thua Thien Hue. Before the start of the feeding experiment, health checks of shrimp were performed by sampling the hepatopancreas of five shrimp directly onto Thiosulfate Citrate Bile Salts (TCBS, Himedia, Mumbai, India) agar and checking for bacterial growth (Phuoc et al., 2021).

A total of 1,200 juveniles with an initial body weight of 0.5(± 0.1) g were distributed randomly into 12 fiberglass tanks (120 L, 3 tanks per diet, 100 shrimp per tank) equipped with continuous aeration. Shrimp were fed four times a day (07:00 h, 12:00 h, 16:00 h, and 20:00 h) with the control or test diets at a rate of 5–7% of wet body weight. During the experimental period, the water parameters were maintained at 27 ± 1°C temperature, pH 7.7–8.3, dissolved oxygen at 5.5–7.3mgL^–1^, salinity at 20–22g L^–1^, and a 12-h light:12-h dark cycle for 60 days. The condition of the tanks was maintained by siphoning off all residual, feces, molts, and dead shrimp in the morning. Uneaten feeds were siphoned after 2 h feeding from tanks. After the 60-day feeding regime, 30 shrimp from each experimental group were collected for the challenge test with *V. parahaemolyticus*. Six shrimp from each group were collected for evaluation of immune responses, including the total hemocyte count, phenoloxidase activity, and lysozyme activity, and 6 other shrimp from each group were used for the phagocytic activity assay.

### Analysis of Growth Performance

At the end of the feeding trial, the juveniles were fasted for 24 h, and then weighed and counted. Based on the weight of each shrimp and the number of juveniles survived, the specific growth rate (SGR), weight gain (WG), feed conversion ratio (FCR), and survival rate (SR) were calculated following the standard formula (Adel et al., 2017):


$$\mathrm{SGR}=\frac{\ln\mathrm{of}\,\mathrm{final}\,\mathrm{weight}\,\left(g\right)-\ln\mathrm{of}\,\mathrm{initial}\,\mathrm{weight}\,\left(g\right)}{\mathrm{number}\,\mathrm{of}\,\mathrm{days}}\times 100$$

$$\mathrm{WG}=\frac{\mathrm{Final}\,\mathrm{weight}\,\left(g\right)-\mathrm{initial}\,\mathrm{weight}\,\left(g\right)}{\mathrm{initial}\,\mathrm{weight}\,\left(g\right)}\times 100$$

$$\mathrm{FCR}=\frac{\mathrm{Feed}\,\mathrm{intake}\,\left(g\right)}{\mathrm{Weight}\,\mathrm{gain}\,\left(g\right)}$$

$$\mathrm{SR}=\frac{\mathrm{Final}\,\mathrm{number}\,\mathrm{of}\,\mathrm{live}\,\mathrm{shrimp}}{\mathrm{Number}\,\mathrm{of}\,\mathrm{initial}\,\mathrm{shrimp}}\times 100$$

### Non-specific Immune Responses Analysis

#### Total Hemocyte Count

The total hemocyte count (THC) was conducted as described by Chiu et al. (2007). Briefly, hemolymph (100 μl) was withdrawn from the ventral sinus of each shrimp using a 1-ml sterile syringe (25-gauge) containing 0.9 ml anticoagulant solution (30 mM trisodium citrate, 0.34 M sodium chloride, and 10 mM EDTA, at a pH of 7.55 and with the osmolality adjusted with glucose to 780 Osm kg^–1^). A drop of each diluted hemolymph sample was placed in a hemocytometer and observed under a light microscope to determine its total hemocyte count.

#### Phenoloxidase Activity

The Phenoloxidase (PO) activity was measured spectrophotometrically by recording the formation of dopachrome produced from l-dihydroxyphenylalanine (l-DOPA). One microliter of diluted hemolymph from each tube was centrifuged at 800 × *g* and 4°C for 20 min. The supernatant was discarded, and the pellet was rinsed, re-suspended gently in 500 μl cacodylate citrate buffer (10 mM sodium cacodylate, 450 mM sodium chloride, and 100 mM trisodium citrate; pH 7.0), and then centrifuged again. The supernatant was discarded, and the pellet was resuspended in 100 μl of cacodylate buffer (10 mM sodium cacodylate, 450 mM sodium chloride, 10 mM calcium chloride, and 260 mM magnesium chloride; pH 7.0), and equal aliquots (cell suspension) were placed into two tubes. One tube was used for measuring the PO activity, and the other tube was measured for background PO activity. The cell suspension (100 μl) was incubated for 10 minat 25–26°C with 50 μl of trypsin (1 mgml^–1^), which served as an elicitor. Fifty microliters of l-DOPA was added, followed by 800 μl of cacodylate buffer 5 min later. The control solution, which consisted of 100 μl of the cell suspension, 50 μl of cacodylate buffer (to replace the trypsin), 50 μl of l-DOPA, and 800 μl of cacodylate buffer, was used for the background PO activity. The shrimp’s PO activity was measured at an OD of 490 nm using a spectrophotometer (Jasco V-630, Hachioji, Tokyo, Japan).

#### Lysozyme Activity

The lysozyme (LYS) activity was quantified following the procedures described by Chiu et al. (2007). Briefly, 500 μl of diluted hemolymph were centrifuged, and the precipitate was mixed with 1 ml (0.02%) of *Micrococcus lysodeikticus* (Sigma, St. Louis, MO, United States). The reaction was carried out at room temperature, and the absorbance at 530 nm was measured after 0.5 and 4.5 min. A unit of LYS activity was defined as the amount of enzyme producing a decrease in absorbance of 0.01 min^–1^, and the specific activity was expressed as U (g protein^–1^).

#### Phagocytic Activity Assay

Twenty microliters of a bacterial suspension of *V. parahaemolyticus* (2 × 10^6^cfuml^–1^) was injected into the ventral sinus. After being injected, the shrimp were kept in separate tanks containing 40L of seawater (20‰) for 2 hat 27 ± 1°C. Hemolymph (100 μl) from six shrimp was withdrawn and mixed with 900 μl of an anticoagulant solution. One hundred microliters of a diluted hemolymph sample was mixed with 100 μl of 0.1% paraformaldehyde and incubated for 30 minat 4°C to fix the hemocytes. Then, 50 μl of the suspension was spread on a glass slide. The slide was placed in a cytospin centrifuge and centrifuged at 113 × *g* for 3 min. The slide was then air-dried, stained with Diff-Quick stain, and observed using a light microscope. Two hundred hemocytes were counted. Phagocytic activity (PA) was expressed as:


$$\mathrm{PA}\left(\%\right)=\frac{\mathrm{Phagocytic}\,\mathrm{hemocytes}}{\mathrm{Total}\,\mathrm{hemocytes}}\times 100$$

### Challenge Test

Pathogenic *V. parahaemolyticus* was obtained from the laboratory of Fish Pathology, Faculty of Fisheries, University of Agriculture and Forestry, Hue University, Vietnam. A single colony of *V. parahaemolyticus* grown on TCBS agar was sub-cultured in 10 ml of Tryptone Soya Broth (Oxoid, Hamsphire, United Kingdom) containing 2% NaCl at 28°C for 24 h to achieve exponential growth. The broth culture was centrifuged at 3,000 rpm for 30 minat 4°C. The supernatant was removed, and the bacterial pellet was re-suspended in saline solution (0.85% NaCl), which corresponds to approximately 10^9^ cfu ml^–1^ for the challenge test. The viable colony counts were performed using the method of Miles et al. (1938).

After 60 days, shrimp fed with different experimental diets (30 shrimp per tank × 3 tanks per treatment = 90 shrimp per treatment) were challenged with *V. parahaemolyticus* by immersion for 1 h. Shrimps were immersed in 10 L of 20‰ seawater containing bacteria at 1.1 × 10^6^ cfu ml^–1^ at 28 ± 1°C, removed after 1 h, and placed into the flow-through experimental tanks (120 L) at 0.38 L min^–1^, with water temperature at 26 ± 2°C for 14 days to record any abnormal behavior, clinical signs, and daily mortality. The bacterial concentration was determined from previous pilot studies and was designed to give 60% total mortalities (data not shown). Aeration was supplied through an air stone to each tank and the shrimp were fed with control diet to apparent satiation twice daily. At the end of the challenge test, the cumulative mortality percentage (%) was calculated as previously described (Kongnum and Hongpattarakere, 2012).

### Statistical Analysis

The data were expressed as mean ± standard deviation. They were first checked for underlying Gaussian distribution of data using a Shapiro–Wilks test. Once this distribution was confirmed, data were analyzed by one-way ANOVA, and multiple comparisons were performed using a Tukey *post hoc* test. All statistical analyses were performed using the SPSS 20.0 (SPSS Inc, Chicago, IL, United States). Differences were considered significant at *p* 0.05.

## Results

### Growth Performances

Growth performances of *L. vannamei* were presented in Table 2. The final body weight, WG, and SGR of shrimp increased in all the groups that were fed with *P. pentosaceus*-supplemented diets when compared with the control diet-fed group. The final body weight and WG of shrimp fed P2 or P3 diet were significantly (*p* < 0.05) higher when compared with the control and P1 groups. These values of shrimp in the group fed with the P1 diet were higher but not significantly different from that of shrimp in the control treatment (*p >* 0.05). The FCR decreased and was significantly lower in all the treatment groups fed with *P. pentosaceus-*supplemented diets compared to that in the control group (*p* < 0.05). Similarly, the survival rate was significantly higher in all the *P. pentosaceus* fed groups when compared with the control diet-fed group. The highest survival rate was observed in P2 treatment where shrimp were fed with *P. pentosaceus*-supplemented diet at the inclusion level of 1 × 10^7^ cfu g^–1^, significantly higher than that observed in P1 treatment where shrimp were fed with *P. pentosaceus* at 1 × 10^6^ cfu g^–1^ (*p* < 0.05) but was not significantly different from that of shrimp fed with *P. pentosaceus* at 1 × 10^8^ cfu g^–1^ (P3) (*p* > 0.05).

**TABLE 2:** Growth performance of shrimp fed with different levels of *P. pentosaceus*.

Parameters	Treatments			
	Control	P1	P2	P3
Initial weight (g shrimp^–1^)	0.53 ± 0.05^a^	0.55 ± 0.03^a^	0.57 ± 0.04^a^	0.58 ± 0.07^a^
Final weight (g shrimp^–1^)	4.1 ± 0.1^a^	4.3 ± 0.3^a^	4.9 ± 0.3^b^	5.0 ± 0.3^b^
Gain weight (%)	677.0 ± 22.1^a^	685.0 ± 23.2^a^	768.0 ± 21.0^b^	770.0 ± 21.0^b^
FCR	1.38 ± 0.16^a^	1.20 ± 0.05^b^	1.05 ± 0.04^c^	1.02 ± 0.03^c^
SGR (% day)	3.41 ± 0.09^a^	3.42 ± 0.07^a^	3.60 ± 0.06^b^	3.60 ± 0.10^b^
Survival (%)	70.0 ± 1.8^a^	75.0 ± 1.6^b^	85.3 ± 1.2^c^	84.7 ± 1.4^c^

*Data are means ± SD of triplicate groups of shrimps. Values in each row with different superscripts are significantly different (p < 0.05). Diets: Control = Basal diet with 0.5% FOS, refer to Table 1; P1: P. pentosaceus at 1 × 10^6^ cfu g^–1^; P2: P. pentosaceus at 1 × 10^7^ cfu g^–1^; P3: P. pentosaceus at 1 × 10^8^ cfu g^–1^.*

### Immune Response

Feeding of diets supplemented with *P. pentosaceus* caused a significant improvement in the immune responses of juvenile *L. vannamei* especially with reference to THC, lysozyme, PO, and phagocyte activity against *V. parahaemolyticus* (Tables 3, 4). A significant increase (*p* < 0.05) in the THC and PO activity was noted in the group fed with *P. pentosaceus*-supplemented diets when compared to the control group. In the case of THC, the highest value was recorded in the P3 group followed by the P2 group, whereas in the case of PO activity, the P3 group recorded maximum PO activity, but was not significantly different from that of the P1 and P2 groups (*p* > 0.05). Feeding of shrimp with a diet supplemented with *P. pentosaceus* in the P2 diet exhibited a maximum increase in the activity of the LYS enzyme. However, no significant difference was recorded with the P3 group (*p* > 0.05). The lysozyme activity of shrimp fed the P1 diet and that of the control group did not differ significantly (*p* > 0.05) (Table 3).

**TABLE 3:** Phenoloxidase (PO) and lysozyme activities, and the total hemocyte count (THC) of shrimp in response to feeding different experimental diets.

Parameters	Treatments			
	Control	P1	P2	P3
THC (× 10^6^ cells ml^–1^)	12.20 ± 0.89^a^	13.80 ± 0.58^b^	14.50 ± 0.85^c^	17.70 ± 1.04*^d^*
PO (100 μl^–1^)	0.19 ± 0.00^a^	0.22 ± 0.01^b^	0.25 ± 0.00^b^	0.26 ± 0.03^b^
Lysozyme (U)	0.66 ± 0.01^a^	0.68 ± 0.04^a^	1.03 ± 0.15^b^	0.94 ± 0.08^b^

*Values are means ± SD of triplicate replicates. Values in the row with different superscripts letters are significantly different (p < 0.05).*

**TABLE 4:** Phagocyte activity of shrimp in treatments with different levels of *P. pentosaceus*.

Parameters	Treatments			
	**Control**	**P1**	**P2**	**P3**
Phagocytic activity (no. of cells)	178.0 ± 2.0^a^	192.0 ± 3.0^b^	196.0 ± 2.0^c^	197.0 ± 2.0^c^
Non-phagocytic activity (no. of cells)	22.0 ± 2.0^a^	8.0 ± 3.0^b^	4.0 ± 2.0^c^	3.0 ± 2.0^c^

*Values are means ± SD of three replicates. Values in the row with different superscripts letters are significantly different (p < 0.05).*

In the present study, the phagocytic activities of shrimp fed *P. pentosaceus*-supplemented diets were significantly higher than the group fed a control diet. Among the treatment groups, those fed with *P. pentosaceus-*supplemented P3 diet exhibited the highest phagocytic activity followed by the P2 and P1 groups. However, there is no significant difference between the P2 and P3 groups (*p* > 0.05) (Table 4).

### Challenge Test

**FIGURE 1:**
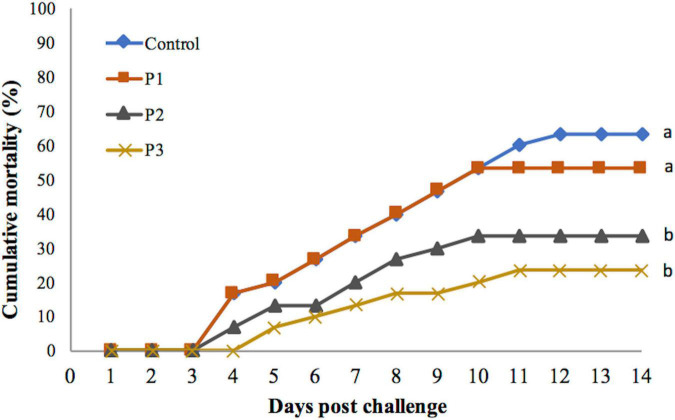
Cumulative mortality percentage of juvenile whiteleg shrimp fed with different doses of *P. pentosaceus* for 60 days and experimentally challenged with *V. parahaemolyticus* for 14 days. Data with different letters are significantly different among groups (*p* < 0.05).

Feeding of diet supplemented with *P. pentosaceus* caused a significant effect on the cumulative mortality percentage of the shrimp, regardless of the dose of *P. pentosaceus* in the feed that was used, noting that isolate was used at its LD_60_ (Figure 1). Mortality was recorded highest in the control group fed with a control diet without *P. pentosaceus* added. The mortality decreased for each treatment as the dose of *P. pentosaceus* increased (Figure 1). The mortality of shrimp fed with the P3 diet was lowest but was not significantly different from the treatment group that was fed with the P2 diet (*p* > 0.05). The mortality of these two treatment groups was significantly lower when compared with the P1 group (*p* < 0.05 for both groups). The mortality of shrimp fed with the P3 diet was lower but was not significantly different from the control group (*p* > 0.05).

Mortality was first observed on day 3 post-challenge in all the experimental groups except in the P3 group that was fed with a diet supplemented with 1 × 10^8^ cfu g^–1^ *P. pentosaceus*. In the P3 group, mortality was noted on day 4 post-challenge. Lower percentage mortality was associated with later onset of mortality. Mortality continued until day 12 post-challenge in the control group. Whereas in the treatment groups, mortality continued only till day 10 except in the P3 group, in which mortality continued until day 11 post-challenge (Figure 1).

## Discussion

Lactic acid bacteria strains have been used as probiotics in aquaculture for a long time, in which *P. pentosaceus* has received more attention in recent years for application in farmed shrimp. It is because of its wide range of beneficial effects, especially on the growth performance, digestive enzyme activity (Adel et al., 2017; Won et al., 2020), immunity, and tolerance towards pathogenic vibrios (Adel et al., 2017). In the present study, we combined the probiotic strain *P. pentosaceus* at different inclusion levels with a fixed level of a known prebiotic component FOS to develop a synbiotic formulation to determine its impact on the growth indices, immune status, and infection stress tolerance trait in commercially important *L. vannamei*. Our results showed that when *P. pentosaceus* was combined with 0.5% FOS especially in the treatment groups P2 and P3, a significant increment in the final weight was achieved. The improvement in the weight gain of the juveniles in response to feeding P2 or P3 diet resulted in an FCR that was significantly lower than for the other diets. It is also noteworthy to mention that in the control group, there was about 63% mortality at the end of the feeding trial. However, in the treatment groups, there was a significant reduction in the mortality, with the P2 and P3 groups having a mortality of 15%. This suggests that the probiotic strain at the inclusion levels of 1 × 10^7^ cfu g^–1^ (P2) or 1 × 10^8^ cfu g^–1^ (P3) and in combination with 0.5% FOS had no adverse effect on the survival of the shrimp juveniles, at least in our described experimental condition, and hence can be considered safe for use in *L. vannamei* feed. While all experimental feeds in the present study are formulated with a prebiotic supplement, the observed improvement in the growth performance was associated with differences in the probiotic component of the synbiotic formulation. To our knowledge, there is no available report on the effect of the dietary supplementation of *P. pentosaceus* in combination with FOS on the growth and survival performances in *L. vannamei* juveniles. However, in earlier studies, Adel et al. (2017) and Won et al. (2020) reported that dietary supplementation of *P. pentosaceus* could cause a significant improvement in the growth performance and nutritional utilization in *L. vannamei*, findings in agreement with that of our study. There are also studies on whiteleg shrimp in which improved growth and feed utilization in response to feeding diets supplemented with probiotics, such as *Bacillus subtilis* (Munaeni et al., 2014; Tsai et al., 2019; Won et al., 2020), *Bacillus licheniformis* (Fan et al., 2021), and *Lactococcus lactis* (Won et al., 2020), were reported. In another study, the use of 0.2–0.4% FOS in shrimp diets was shown to improve the growth performance, FCR, and the microbial diversity, and to suppress several potential pathogens, such as *Vibrio tubiashii, V. parahaemolyticus*, and *Photobacterium damselae*-like strains in the intestine of whiteleg shrimp (Hu et al., 2019). However, it is noteworthy to mention that dietary supplementation of FOS at 0, 0.15, and 0.30% did not cause any significant beneficial effect on the survival, weight gain, and immune responses of the shrimp juveniles (Mustafa et al., 2020). Previous studies reported that combining prebiotics, such as MOS, FOS, or galactooligosaccharide (GOS), with probiotic bacteria, *Enterococcus* spp., *Bacillus* spp., or LAB strains improved nutrient utilization (Zhou et al., 2009; Won et al., 2020) and growth performance in shrimps by increasing the absorptive surface area of intestinal microvilli (Das et al., 2017; Huynh et al., 2017; Chen et al., 2020; Butt et al., 2021; Yao et al., 2021). During synbiotic fermentation, many essential amino acids are released, such as histidine, isoleucine, leucine, lysine, tryptophan, and nonessential amino acids such as alanine, glutamate, and tyrosine (Ndagijimana et al., 2009; Rodrigues et al., 2011), and vitamins, such as ascorbate (vitamin C), folate (vitamin B9), and cyanocobalamin (vitamin B12) (Rossi et al., 2005; Rodrigues et al., 2011). These biologically active compounds might play an important role in the food assimilation, absorption, and growth of aquatic animals. Besides, the positive effect of the conjoint administration of FOS and *P. pentosaceus*, in the form of synbiotics, on the growth performances of whiteleg shrimp could be due to an increase in the digestive enzyme activities induced by the *P. pentosaceus*. The presence of this LAB strain might stimulate the production of endogenous enzymes in the host or contribute to the total enzyme activity of the gut as reported previously in shrimps (Adel et al., 2017; Wanna et al., 2021).

Several lines of evidence suggested that the crustacea hemocytes, PO, and lysozyme activity play an important role in generating a protective immune response against bacterial diseases including those caused by vibrios (Oktaviana et al., 2014; Zubaidah et al., 2015; Kuo et al., 2021). Shrimps possess three hemocyte types named hyaline cells, semigranular cells, and granular cells (Johansson et al., 2000). Hemocyte count can vary greatly in response to infection, environmental stress, and the life cycle of the animal (Zubaidah et al., 2015; Kuo et al., 2021). An increase in the THC provides enhanced immune capability during a period of stress leading to disease resistance in crustacean (Chiu et al., 2007). The prophenoloxidase plays an important role in eliciting protective immune responses in the crustaceans. The proposed mechanism of prophenoloxidase is that active PO induces oxidation of phenols to quinones, and results in the production of melanin, which can hold and barricade infectious pathogens leading to induced phagocytosis, and cytotoxic reactant production (Chiu et al., 2007; Amparyup et al., 2012). In the current study, the supplemental *P. pentosaceus* at the inclusion level 1 × 10^6^ cfu g^–1^ (P1), or 1 × 10^7^ cfu g^–1^ (P2), or 1 × 10^8^ cfu g^–1^ (P3) in the diets with 0.5% FOS induced the most significant improvement in PO activity, compared to the control diets. Phagocytosis is an important cellular defense mechanism, whereas lysozyme is an important humoral defense mechanism in crustaceans. Results of the present study are in line with previous studies that demonstrated that synbiotic increased proliferation of hemocytes and activity of PO and lysozyme enzymes (Adel et al., 2017). Our results suggest that the improvement in the tested immune effector molecules in the group fed with a diet supplemented with 1 × 10^7^ cfu g^–1^ (P2) or 1 × 10^8^ cfu g^–1^ (P3) was strongly associated with a significant reduction in the mortality of the *V. parahemolyticus*-challenged *L. vannamei* shrimp fed with *P. pentosaceus*-supplemented diets. This result suggests that besides having a positive effect on the growth, the probiont *P. pentosaceus* have additional health-beneficial effects.

In the gut, bacteria can adhere to the intestinal epithelial surfaces by specific attachment of bacterial surface proteins to complementary oligosaccharides on the tissue surface. The oligosaccharides such as FOS can modulate the adhesive of selective bacteria by acting as antiadhesion agents to opportunistic pathogens (Van den Abbeele et al., 2009; Altamimi et al., 2016). Moreover, oligosaccharides can produce short-chain fatty acids (SCFA) that lower intestinal pH in the colon, consequently creating an unsuitable condition for the growth and survival of pathogenic bacteria (Chen et al., 2020). This evidence might explain for higher survival of the *L. vannamei* that was observed in the treatment groups (P2 and P3) after being challenged with *V. parahaemolyticus* when *P. pentosaceus* was supplemented in the basal diet with FOS.

In conclusion, a synbiotic formulation was developed combining *P. pentosaceus* at the inclusion level of 1 × 10^7^ cfu g^–1^ or 1 × 10^8^ cfu g^–1^ with 0.5% FOS, exhibiting a beneficial effect on the growth performance and immune response of whiteleg shrimp. Our results also showed that an improvement in the resistance of the juveniles towards *V. parahaemolyticus* challenge was associated with a significant improvement in the immune response as manifested by an increase in the activity of LYS and PO enzymes and hemocyte counts in the synbiotic fed groups. Overall results suggest that a combination of *P. pentosaceus* and FOS can be considered as a potential synbiotic for farmed shrimp species.

## Data Availability Statement

The original contributions presented in the study are included in the article/Supplementary Material, further inquiries can be directed to the corresponding author/s.

## Ethics Statement

The Animal Ethics Committee at University of Agriculture and Forestry, Hue University was not established during the time this study was conducted. In the absence of a regulatory framework for formal ethical approval, the work was conducted according to the ethical standards of the UK Home Office, based on training received by the corresponding author at the Institute of Aquaculture, University of Stirling, Stirling, United Kingdom.

## Author Contributions

NH, DT, KB, and NP conceived the study. NH and NL conducted the challenge experiments. NH acquired the funding. NP, NH, and KB wrote the manuscript. All authors read and approved the final manuscript.

## Conflict of Interest

The authors declare that the research was conducted in the absence of any commercial or financial relationships that could be construed as a potential conflict of interest.

## Publisher’s Note

All claims expressed in this article are solely those of the authors and do not necessarily represent those of their affiliated organizations, or those of the publisher, the editors and the reviewers. Any product that may be evaluated in this article, or claim that may be made by its manufacturer, is not guaranteed or endorsed by the publisher.
