# Supplementary material for: The Combined Use of Pediococcus pentosaceus and Fructooligosaccharide Improves Growth Performance, Immune Response, and Resistance of Whiteleg Shrimp Litopenaeus vannamei Against Vibrio parahaemolyticus
Source: Front Microbiol. 2022 Feb 25;13:826151. doi: 10.3389/fmicb.2022.826151 (PMC8914372; doi:10.3389/fmicb.2022.826151)
Supplement: Supplementary file 1 [file Table_1.pdf]

Table Antagonistic effect to *Vibrio parahaemolyticus* and non-hemolytic on whiteleg shrimp blood agar of the six isolates of *Pediococcus pentosaceu*s

No.	Strain	Heamolysis	Inhabitation zone (mm)
1	*Pediococcus pentosaceu*s R1	Non- heamolysis	2-4
2	*Pediococcus pentosaceu*s R2	Non- heamolysis	3-5
3	*Pediococcus pentosaceu*s R3	Non- heamolysis	4-6
4	*Pediococcus pentosaceu*s R4	Non- heamolysis	6-8
5	*Pediococcus pentosaceu*s R5	Non- heamolysis	5-7
6	*Pediococcus pentosaceu*s R6	Non- heamolysis	10-12
